# The value of ECG changes in risk stratification of COVID‐19 patients

**DOI:** 10.1111/anec.12815

**Published:** 2021-01-29

**Authors:** Luca Bergamaschi, Emanuela Concetta D’Angelo, Pasquale Paolisso, Sebastiano Toniolo, Michele Fabrizio, Francesco Angeli, Francesco Donati, Ilenia Magnani, Andrea Rinaldi, Lorenzo Bartoli, Chiara Chiti, Mauro Biffi, Carmine Pizzi, Pierluigi Viale, Nazzareno Galié

**Affiliations:** ^1^ Unit of Cardiology Department of Experimental, Diagnostic and Specialty Medicine‐DIMES University of Bologna Bologna Italy; ^2^ Unit of Infectious Diseases Department of Medical and Surgical Sciences S. Orsola Hospital University of Bologna Bologna Italy

**Keywords:** cardiac injury, COVID‐19, electrocardiogram, outcomes, risk stratification

## Abstract

**Background:**

There is growing evidence of cardiac injury in COVID‐19. Our purpose was to assess the prognostic value of serial electrocardiograms in COVID‐19 patients.

**Methods:**

We evaluated 269 consecutive patients admitted to our center with confirmed SARS‐CoV‐2 infection. ECGs available at admission and after 1 week from hospitalization were assessed. We evaluated the correlation between ECGs findings and major adverse events (MAE) as the composite of intra‐hospital all‐cause mortality or need for invasive mechanical ventilation. Abnormal ECGs were defined if primary ST‐T segment alterations, left ventricular hypertrophy, tachy or bradyarrhythmias and any new AV, bundle blocks or significant morphology alterations (e.g., new Q pathological waves) were present.

**Results:**

Abnormal ECG at admission (106/216) and elevated baseline troponin values were more common in patients who developed MAE (*p* = .04 and *p* = .02, respectively). Concerning ECGs recorded after 7 days (159), abnormal findings were reported in 53.5% of patients and they were more frequent in those with MAE (*p* = .001). Among abnormal ECGs, ischemic alterations and left ventricular hypertrophy were significantly associated with a higher MAE rate. The multivariable analysis showed that the presence of abnormal ECG at 7 days of hospitalization was an independent predictor of MAE (HR 3.2; 95% CI 1.2–8.7; *p* = .02). Furthermore, patients with abnormal ECG at 7 days more often required transfer to the intensive care unit (*p* = .01) or renal replacement therapy (*p* = .04).

**Conclusions:**

Patients with COVID‐19 should receive ECG at admission but also during their hospital stay. Indeed, electrocardiographic alterations during hospitalization are associated with MAE and infection severity.

## 1 INTRODUCTION

Coronavirus disease 2019 (COVID‐19) is a global pandemic, as World Health Organization (WHO) declared on March 11, 2020 (Mahase, 2020). COVID‐19 is caused by the Severe Acute Respiratory Syndrome Coronavirus‐2 (SARS‐CoV‐2): this single‐stranded enveloped RNA virus interacts through binding of surface spike protein human angiotensin‐converting enzyme 2 (ACE2) receptor. ACE2 is expressed in the lung and intestinal epithelium, vascular endothelium, kidneys, and heart as well (Tikellis & Thomas, 2012). The exact mechanism of cardiac involvement remains unclear. Probably, it is not only related to the interaction between protein and receptor. Other suggested mechanisms of COVID‐19 related to cardiac involvement include cytokine storm and hypoxia inducing excessive intracellular calcium leading to cardiac myocyte apoptosis (Zheng et al., 2020). Myocardial injury was found among early cases in China. Previous studies had confirmed that cardiac injury (elevated high‐sensitivity Troponin I or new ECG or echocardiographic abnormalities) was present in 7 to 1% of patients overall, and 26% required intensive care (Wang et al., 2020). A recent study of Shi et al. comparing 82 COVID‐19 patients with and without cardiac injury concluded that cardiac injury is associated with a high risk of in‐hospital mortality (Shi et al., 2020). ECG changes during viral infections were previously studied: ECG abnormalities during the 2009 H1N1 influenza infection were transient and not correlated with preexisting patient characteristics or with outcomes (Akritidis et al., 2010). No specific ECG changes have been described in patients with SARS‐CoV2 infection yet. The real prevalence of ECG anomalies and the incidence of benign and malignant arrhythmias in COVID‐19 infection is still not well defined (Kochi et al., 2020).

This study aims to investigate the relationship between abnormal serial ECG findings in patients with COVID‐19 and major adverse events (MAE), considered as the composite of all‐cause intra‐hospital mortality or respiratory failure requiring orotracheal intubation (OTI), to define their prognostic value.

## 2 METHODS

### 2.1 Study subjects and design

The study enrolled 269 consecutive patients admitted to Sant’Orsola ‐ Malpighi Hospital of Bologna University with laboratory‐confirmed SARS‐CoV‐2 infection and radiological findings suggestive of interstitial pneumonia from March 01, 2020 to April 10, 2020. Patients were followed up until April 20, 2020. The diagnosis of COVID‐19 was established according to the WHO interim guidance and confirmed by RNA detection of the SARS‐CoV‐2 in the clinical laboratory of Bologna Hospital (Paolisso et al., 2020). Chest radiographs or computed tomography (CT) scans were also done for all inpatients to assess lung parenchyma involvement. The exclusion criteria were age under 18 years old, lack of ECG at admission, and interstitial pneumonia without microbiological confirmation of SARS‐CoV‐2.

Intra‐hospital all‐cause mortality, respiratory failure requiring orotracheal intubation, admission to intensive care unit (ICU), acute kidney injury treated with renal replacement therapy, and need for extracorporeal membrane oxygenation (ECMO) were collected. We considered major adverse event (MAE) the composite of all‐cause death and respiratory failure requiring orotracheal intubation during the hospitalization.

Written informed consent was waived by the designated hospital's ethics committee for patients with emerging infectious diseases.

### 2.2 Electrocardiographic analysis

All 12‐lead electrocardiograms recorded on admission and 7 days later (paper speed of 25 mm/s and 1 millivolt equivalent to 10 mm) were analyzed off‐line. According to pre‐defined criteria, two expert cardiologists blinded to clinical information and non‐invasive diagnostic results, independently evaluated the ECG. A third consensus re‐evaluation resolved disagreements in qualitative evaluations.

The considered parameters were as follows: rhythm (sinus, supraventricular or ventricular arrhythmias); heart rate; PR and QRS durations; QT and QTcorrected (using Bazett or Fridericia formula when the heart rate was >90 beats per minute was used) intervals; conduction disturbances (atrioventricular block, bundle branch block, or fascicular block) and ST‐T segment alterations.

The ST‐segment deviation was measured as the height difference (in millimeters) between the J point and the isoelectric line (TP segment). T wave was analyzed in all 12 leads and classified as normal (positive in all leads apart from III, aVR, V1, with voltage ≥0.1 mV), inverted (negative in any lead except III, aVR, V1, with voltage ≥0.1 mV) or flat (voltage <0.1 mV). Pathological Q waves were identified as any Q wave >40 ms wide, >2 mm deep or >25% of QRS complex depth.

According to the ESC guidelines (Ibanez et al., 2018; Roffi et al., 2016), ST‐T segment alterations were classified as primary if suggesting acute ischemia. Left ventricular hypertrophy (LVH) was defined using Sokolow–Lyon criteria (S in V1 + R in V5 or V6 ≥35 mm or R in aVL ≥11 mm) with or without secondary ST‐T segment changes.

Electrocardiogram was defined as abnormal for any patient if ischemia alterations, left ventricular hypertrophy, tachy or bradyarrhythmias, and any new atrioventricular (AV), bundle branch blocks, or significant morphology alterations (e.g., new Q pathological waves) were present. Otherwise, patients presenting sinus rhythm without previously described alterations were reported as normal.

### 2.3 Clinical data

We collected cardiac biomarkers, troponin I (Tn I), creatine kinase (CK), and brain natriuretic peptide (BNP). Troponin I levels were defined as elevated if they were above the "high‐sensitive" assay‐specific upper reference limit (cut‐off of 11.6 ng/L for women and 19.8 ng/L for men). Demographic characteristics (age and sex), clinical data (symptoms, comorbidities, laboratory findings) and therapy were collected from electronic medical records. Severe COVID‐19 was defined as meeting arterial oxygen saturation ≤93% at rest or PaO_2_/FiO_2_ ≤300 mm Hg. We did not include respiratory rate ≥30 breaths/min according to the Diagnosis and Treatment Plan of COVID‐19 suggested by National Health Commission of China due to the considerable inter‐observer variability (Yang et al., 2020).

### 2.4 Statistical analyses

Continuous and categorical variables were presented as median (IQR) and *n* (%), respectively. Pearson χ^2^ test was used to compare categorical patient characteristics among groups. The normal distribution of continuous variables was assessed using the Shapiro–Wilks test and the equality of variance was tested between groups using Levene's test. In the case of departure from normality, non‐parametric tests (Mann–Whitney *U* test) were used in the normality distribution of values, parametric tests (Fisher's exact test) to compare differences between the two groups were performed. To explore predictors of major adverse events, a multivariable Cox regression was performed. Statistical analyses were performed using IBM SPSS, version 25.

## 3 RESULTS

**FIGURE 1:**
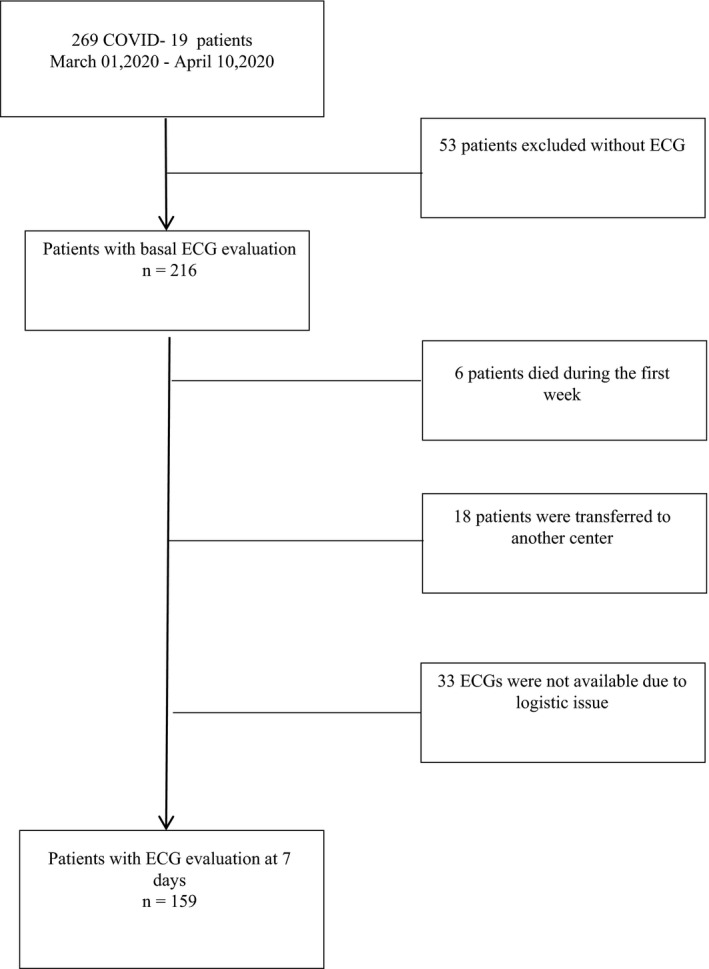
Flow‐chart. Flow‐chart of the enrollment of COVID‐19 patients with serial ECG evaluations. ECG, electrocardiogram

Out of 269 consecutive patients with confirmed COVID‐19 diagnosis, 53 patients were excluded because they did not have ECGs at admission (Figure 1). The final sample, therefore, included 216 patients. Overall, the study population's mean age was 67.0 (IQR 56.75–79.0) years old and 66% were male. Fifty‐eight patients had no medical history, 59.3% were hypertensive and the median hospital length of stay was 11.0 (IQR 7.0–16.5) days. Two hundred and sixteen patients had ECG at admission; however, 7‐day ECG was recorded in 159 patients (73.6%).

### 3.1 ECG findings at admission and after 7 days

Admission ECG characteristics are shown in Table 1. Sinus rhythm was present in 89.8%, whereas atrial fibrillation in 9.3%. The median heart rate was 78.0 (IQR 69.5–89.0) bpm. About 5.5% of patients had first‐degree atrioventricular block, and 18.5% had intraventricular conduction anomalies (left anterior fascicular block, right or left bundle blocks). The median QRS duration was 93.0 (IQR 85.0–105.0) msec, and the median QTc interval was 440.0 (IQR 422.0–465.0) msec.

**TABLE 1:** Electrocardiogram findings and outcomes of COVID‐19 patients at admission

	Total *N* = 216	No major events *N* = 162	Major events *N* = 54	*p*‐value
ECG evaluation				
Sinus rhythm	194 (89.8%)	143 (88.3%)	51 (94.4%)	.2
Atrial fibrillation	20 (9.3%)	17 (10.5%)	3 (5.6%)	.3
HR, bpm	78 (69–89)	77 (69–88)	81 (70–96)	.3
First‐degree AV block	12 (5.5%)	9 (5.5%)	3 (5.8%)	.9
QRS complex, msec	93 (85–105)	92 (85–104)	95.5 (87–105)	.3
Peripheral low voltage	12 (5.6%)	8 (4.9%)	4 (7.4%)	.5
LAFB	19 (8.8%)	11 (6.8%)	8 (14.8%)	.07
RBBB	15 (6.9%)	9 (5.6%)	6 (11.1%)	.1
LBBB	6 (2.8%)	4 (2.5%)	2 (3.7%)	.6
QT, msec	395 (360–428)	395 (360–428)	400 (360–428)	.8
QTc, msec	440 (422–465)	439 (420–460)	460 (430–473)	.05
Normal ECG	110 (50.9%)	89 (54.9%)	21 (38.9%)	.04
Abnormal ECG	106 (49.1%)	73 (45.1%)	33 (61.1%)	
Primary ST‐T segment alterations	12 (5.6%)	5 (3.1%)	7 (13.0%)	.006
Left ventricular hypertrophy	16 (7.4%)	6 (3.7%)	10 (18.5%)	<.001
Other ECG findings	78 (36.1%)	61 (37.7%)	17 (31.5%)	.4
Elevated hs‐Tn I	26/60 (43.3%)	10 (30.3%)	16 (59.3%)	.02

Note

Continuous variables are presented as median (IQR) while categorical ones as *n* (%) or *n*/*N* (%), where *N* is the total number of patients with available data.

Abbreviations: AV, atrioventricular; ECG, electrocardiogram; HR, heart rate; hs – Tn I, high‐sensitivity cardiac Troponin I; LAFB, left anterior fascicular block; LBBB, left bundle branch block; RBBB, right bundle branch block.

Other findings = new tachy or bradyarrhythmias and any new AV, bundle blocks or significant morphology alterations (e.g., new Q pathological wave)

At admission, ECG was normal in 110 patients (50.9%), whereas 106 patients (49.1%) had an abnormal ECG.

Concerning 7‐day ECG, abnormal alterations were reported in 85 out of 159 (53.5%) patients. Sinus rhythm was present in 140 patients (88.1%) and atrial fibrillation in 17 (10.7%). The median heart rate was 76.0 (IQR 65.0–84.25) bpm and QTc interval was 452.0 (IQR 432.0–475.0) msec. The median QRS duration was 94.0 (IQR 87.0–104.0) msec. Table 2 shows other findings of 7‐day ECG.

**TABLE 2:** Electrocardiogram findings and outcomes of COVID‐19 patients at 7 days of hospitalization

	Total *N* = 159	No major events *N* = 111	Major events *N* = 48	*p*‐value
ECG evaluation				
Sinus rhythm	140 (88.1%)	96 (86.5%)	44 (91.7%)	.3
Atrial fibrillation	17 (10.7%)	13 (11.7%)	4 (8.3%)	.5
HR, bpm (IQR)	76 (65–84)	74 (65–80)	82 (73–95)	.001
First‐degree AV block	7 (4.4%)	4 (3.6%)	3 (6.3%)	.4
QRS complex, msec	94 (87–104)	92 (86–104)	96 (90–103)	.2
Peripheral low voltage	8 (5.0%)	3 (2.7%)	5 (10.4%)	.04
LAFB	12 (7.5%)	8 (7.2%)	4 (8.3%)	.8
RBBB	11 (6.9%)	7 (6.3%)	4 (8.3%)	.6
LBBB	5 (3.1%)	3 (2.7%)	2 (4.2%)	.6
QT, msec	410 (380–440)	417 (385–440)	398 (366–440)	.09
QTc, msec	452 (432–475)	450 (433–474)	458 (431–484)	.4
Normal ECG	74 (46.5%)	61 (55.0%)	13 (27.1%)	.001
Abnormal ECG	85 (53.5%)	50 (45.0%)	35 (72.9%)	
Primary ST‐T segment alterations	13 (8.2%)	5 (4.5%)	8 (16.7%)	.01
Left ventricular hypertrophy	14 (8.8%)	6 (5.4%)	8 (16.7%)	.02
Other ECG findings	58 (36.5%)	39 (35.1%)	19 (39.6%)	.6
Delta HR ≥20%^a^	17/149 (11.4%)	7 (6.3%)	10 (26.3%)	.001
Wide QRS acquired^b^	6/146 (4.1%)	1 (1%)	5 (11.6%)	.003

Note

Continuous variables are presented as median (IQR) while categorical ones as *n* (%) or *n*/*N* (%), where *N* is the total number of patients with available data.

Abbreviations: AV, atrioventricular; ECG, electrocardiogram; HR, heart rate; LAFB, Left anterior fascicular block; LBBB, Left bundle branch block; RBBB, right bundle branch block.

Other findings, new tachy or bradyarrhythmias and any new AV, bundle blocks or significant morphology alterations (e.g., new Q pathological wave).

^a^ Respect to admission heart rate.

^b^ Respect to admission QRS duration.

### 3.2 ECG findings and outcomes

Table 1 compared the ECG findings at admission in patients with or without major adverse events. Abnormal ECG at admission and elevated baseline troponin I values were more common features in patients who developed MAE (61.1% versus 45.1%; *p*‐value = .04 and 59.3% versus 30.3%; *p*‐value = .02, respectively).

**FIGURE 2:**
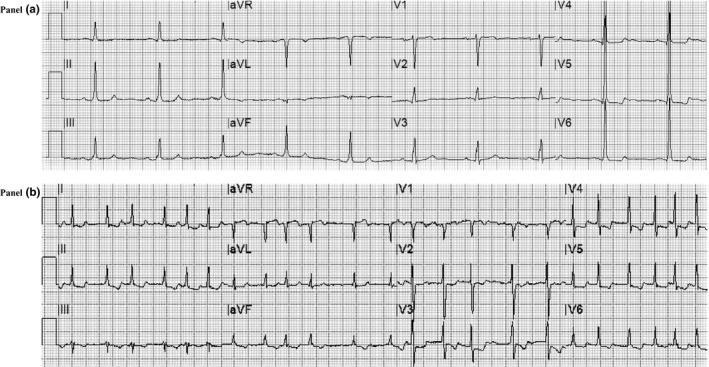
Electrocardiogram alterations correlated with major adverse events in COVID‐19. *Panel A*. Basal ECG of a patient with severe COVID‐19 with signs of left ventricular hypertrophy (according to Sokolow–Lyon criteria with secondary ST‐T segment alterations) who required orotracheal intubation for worsening of respiratory failure with subsequent recovery. *Panel B*. ECG recorded at 7 days of hospitalization showing atrial fibrillation with high ventricular rate response and ischemic alterations (primary ST‐T segment alterations) in a patient who died due to respiratory failure at 16 days of hospitalization

Among admission ECG, abnormalities ischemic alterations with primary ST‐T segment alterations (13.0% versus 3.1% *p*‐value = .006) and signs of left ventricular hypertrophy (18.5 versus 3.7%; *p*‐value = <.001) were associated with worse prognosis (Figure 2).

After 1 week, the correlation between ECG findings and MAE was even stronger than at admission. Patients with MAE had more frequently low peripheral voltages (10.4% versus. 2.7%; *p*‐value = .04) and higher heart rate (*p*‐value = .001). Moreover, compared to baseline values, increase of at least 20% in heart rate (26.3% versus 6.3%; *p*‐value = .001) or in QRS duration (11.6% versus 1.0%; *p*‐value = .003) significantly correlated with worse outcomes.

Also, at 7‐day ECG, patients with MAE had more frequently abnormal ECG as compared to patients with a better prognosis (72.9% versus 45.0% *p*‐value = .001). All‐cause mortality (25.9% versus 7.0%, *p*‐value = .002), admission to intensive care (28.2% versus 12.2%, *p*‐value = .01), and acute renal failure necessitating renal replacement therapy (8.2% versus 1.3%, *p*‐value = .04) were more frequently in patients with abnormal 7–day ECG, as shown in Table 3.

**TABLE 3:** All‐cause intra‐hospital mortality or orotracheal intubation and other outcomes according to ECG findings at 7 days of hospitalization

	Total *N* = 159	Normal ECG *N* = 74	Abnormal ECG *N* = 85	*p*‐value
All‐cause mortality/OTI	48 (30.2%)	13 (17.6%)	35 (41.2%	.001
All‐cause mortality	26/152^a^ (17.1%)	5 (7.0%)	21 (25.9%)	.002
Admission to ICU	33 (20.8%)	9 (12.2%)	24 (28.2%)	.01
CVVH	5 (3.1%)	1 (1.3%)	7 (8.2%)	.04
ECMO	4 (2.5%)	1 (1.4%)	3 (3.5%)	.4

Note

Categorical variables are presented as *n* (%) or *n*/*N* (%), where *N* is the total number of patients with available data.

Abbreviations: CVVH, renal failure requiring continuous veno‐venous hemofiltration; ECMO, extracorporeal membrane oxygenation; ICU, intensive care unit; OTI, orotracheal intubation.

^a^ 7 patients were still hospitalized.

Among all the pathological changes, primary ST‐T segment alterations and left ventricular hypertrophy were still significantly correlated with MAE (Figure 2). During the hospitalization, new onset of atrial fibrillation was associated with a higher incidence of MAE (12.9% versus 4.1%; *p*‐value = .001). Nevertheless, no differences were found in history or presence of AF at baseline. The gender did not affect ECG presentation at either baseline or 7 days of hospitalization.

### 3.3 Clinical findings in normal and abnormal 7‐day ECG

In Table 4, we reported baseline and clinical findings in 74 patients with normal ECG compared to 85 patients with abnormal ECG evaluated at 7‐day ECG.

**TABLE 4:** Baseline demographic, clinical, laboratory findings and specific treatments of COVID‐19 patients with normal or pathologic ECG at 7 days

	Total *N* = 159	Normal ECG *N* = 74	Abnormal ECG *N* = 85	*p*‐value
Age, years	67 (57–79)	63 (54–75)	73 (62–85)	.001
Male gender	105 (66.0%)	49 (66.2%)	56 (65.9%)	.9
BMI, Kg/m^2^	27.3 (25.1–30.25)	26.2 (24.32–30.82)	27.3 (25.4–31.2)	.3
Cardiovascular risk factors				
Current/past smoking	58 (36.5%)	29 (39.1%)	29 (34.Q%)	.5
Hypertension	95 (59.7%)	36 (48.6%)	59 (69.4%)	.01
Dyslipidemia	38 (23.9%)	16 (21.6%)	22 (25.9%)	.5
Type−2 diabetes	24 (15%)	12 (16.2%)	12 (14.1%)	.7
Medical history				
Previous AMI	13 (8.2%)	3 (4%)	10 (11.8%)	.08
COPD	26 (16.3%)	12 (16.2%)	14 (16.5%)	.96
CKD	14 (8.8%)	6 (8.1%)	8 (9.4%)	.8
Stroke/TIA	9 (5.7%)	4 (5.4%)	5 (5.9%)	.9
Atrial fibrillation	20 (12.6%)	6 (8.1%)	14 (16.4%)	.1
Clinical presentation				
SBP	120 (110–134)	125 (112–135)	125 (110–135)	.3
DBP	70 (70–80)	75 (70–85)	70 (60–80)	.08
Fever (> 37.3 C°)	130/153 (85.0%)	63 (88.7%)	67 (81.7%)	.2
Cough	101/153 (66.0%)	47 (66.2%)	54 (65.9%)	.9
Dyspnea	88/153 (57.5%)	36 (50.7%)	52 (63.4%)	.1
Laboratory parameters				
Hemoglobin, g/dl	12.8 (11.3–14.3)	13.2 (11.35–14.22)	12.2 (10.8–14.0)	.1
White blood cells, *N*/µl	6,900 (5,000–9689)	5,990 (5,015–9,145)	7,425 (5192–11,342)	.1
Lymphocyte, %	17 (10–25)	17 (11–25)	14 (8–23)	.06
Platelet, x10^9^ per L	204 (154–268)	195 (165–246)	210 (149–278)	.5
Creatinine, mg/dl	0.9 (0.73–1.14)	0.85 (0.71–1.05)	0.95 (0.75–1.3)	.05
Blood glucose, mg/dl	105 (91–136)	105 (89–121)	110 (96–145)	.1
ALT, U/L	27 (16–41)	27 (19–41)	24 (16.0–38.25)	.7
AST, U/L	34 (24–48)	34 (23–52)	34 (26–48)	.5
LDH, U/L	308 (232–400)	283 (243–368)	347 (263–407)	.03
Creatine kinase, U/L	80 (48–195)	93 (46–264)	84 (50–184)	.8
C reactive protein, mg/dl	6.62 (2.62–13.97)	7.7 (2.77–16.0)	8.6 (3.7–16.1)	.3
Interleukin−6, pg/ml	26.8 (13.0–75.6)	22.8 (14.8–52.3)	32.7 (13.5–100.0)	.3
D‐dimer, μg/ml	0.7 (0.52–1.50)	0.64 (0.48–1.71)	1.2 (0.52–1.66)	.4
Procalcitonin, ng/ml	0.15 (0.10–0.60)	0.10 (0.10–0.90)	0.20 (0.10–1.60)	.08
BNP, pg/dl	260 (34–550)	38 (10–117.7)	416 (116–1,160)	.03
Arterial blood gas				
Body temperature, °C	37.6 (37.0–38.0)	37.7 (37.0–38.0)	37.5 (367–38.0)	.2
SpO_2_	96 (94–98)	96 (94–97)	96 (93–98)	.8
PaO_2_	72 (63–85)	74 (65–85)	69 (60–90)	.4
FiO_2_	21 (21–31)	21 (21–26)	28 (21–50)	<.001
PaO_2_/FiO_2_ ratio	302 (210–357)	340 (259–395)	241 (173–319)	<.001
Severe COVID−19	80 (50.3%)	27 (36.4%)	53 (62.3%)	.001
Therapy				
Hydroxychloroquine	123 (77.3%)	57 (77%)	66 (77.6%)	.9
Tocilizumab	24 (15%)	14 (18.9%)	10 (11.7%)	.2
β‐lactam antibiotics	102 (64.1%)	46 (62.2%)	56 (65.9%)	.6
Macrolide antibiotics	78 (49%)	35 (47.3%)	43 (50.6%)	.7

Note

Continuous variables are presented as median (IQR) while categorical ones as *n* (%) or *n*/*N* (%), where *N* is the total number of patients with available data.

Abbreviations: ALT, alanine aminotransferase; AMI, acute myocardial infarction; AST, aspartate aminotransferase; BMI, body max index; BNP, Brain natriuretic peptide; CKD, chronic kidney disease; COPD, chronic obstructive lung disease; DBP, diastolic blood pressure; FiO_2_, fraction of inspired oxygen. Severe COVID‐19; LDH, lactate dehydrogenase; PaO_2_, arterial partial pressure of oxygen; SBP, systolic blood pressure; SpO_2_ ≤93% or PaO_2_/FiO_2_ ratio ≤300.

Abnormal ECG was associated with older age (*p*‐value = .001), history of arterial hypertension (*p*‐value = .01), higher lactate dehydrogenase (LDH), and BNP values (*p*‐value = .03). In contrast, there were no differences in other cardiovascular risk factors, medical history, clinical presentation, medical therapy, and other main laboratory findings among the two groups.

As expected, 7‐day ECG alterations were more frequently associated with higher FiO_2_ (*p*‐value <.001) and lower PaO_2_/FiO_2_ ratio (*p*‐value <.001). Noteworthy, the prevalence of severe COVID‐19 was significantly higher in patients with abnormal ECG (62.3% versus 36.4%; *p*‐value = .001) compared to others.

Finally, a multivariable Cox regression was performed to analyze the predictors of MAE during hospitalization (Table 5). The multivariable analysis, adjusted for age, gender, and history of hypertension showed that the presence of pathological 7‐day ECG (HR 3.2; 95% CI 1.2–8.7; *p*‐value = .02) besides basal PaO_2_/FiO_2_ ratio ≤300 (HR 6.1; 95% CI 2.04–18.2; *p*‐value = .001) was independent predictors of MAE in patients with COVID‐19.

**TABLE 5:** Predictors of all‐cause intra‐hospital mortality or orotracheal intubation

	HR	95% CI	*p*‐value
Age, years	0.98	0.95–1.02	.4
Female sex	0.7	0.27–1.79	.5
Hypertension	1.2	0.4–3.4	.7
PaO_2_/FiO_2_ ratio ≤300	6.1	2.04–18.2	.001
Abnormal 7‐days ECG	3.23	1.2–8.7	.02

Abbreviations: FiO_2_, fraction of inspired oxygen; PaO_2_, arterial partial pressure of oxygen.

## 4 DISCUSSION

This study evaluated the ECG findings at admission and after 7 days of hospitalization to predict the prognosis in SARS‐CoV‐2 hospitalized patients. Our data showed that ECG is useful in identifying patients with a worse in‐hospital clinical outcome. Repeated ECG at 7 days can be a strong clinical tool to evaluate the adverse in‐hospital outcome.

In our cohort, there was a significant association between abnormal ECG and major adverse events in patients with COVID‐19. The abnormal ECG findings at admission, as a marker of heart disease, identify subgroups of patients with COVID‐19 at a greater risk for adverse prognosis during the hospital stay. Therefore, serial ECG could help clinicians stratify the overall risk in COVID‐19 patients, given the association between 7‐day ECG alterations and the higher rate of intensive care, orotracheal intubation, and renal replacement therapy and death.

### 4.1 ECG alterations and cardiac involvement in COVID‐19

According to the recent literature on COVID‐19, severe respiratory distress was independently associated with the need for intensive care and intra‐hospital mortality (Yang et al., 2020). New perspectives enlightened that SARS‐CoV‐2 infection is associated with cardiac injury resulting from a direct or indirect effect on cardiovascular system, as described in other coronavirus strains (Inciardi et al., 2020; Shi et al., 2020). ECG is a useful tool in everyday practice to recognize myocardial damage due to the widespread availability. Interestingly, abnormal changes at 12‐lead electrocardiogram did not reflect the population's baseline clinical characteristics but were directly associated with the severity of COVID‐19. In fact, there were no differences in clinical history, hemodynamic state, main laboratory parameters, and medical treatments whether patients had normal 7‐day ECG or not. This underlines the independent role of serial ECG to identify the worsening of respiratory infectious disease.

Specific ECG changes were related to major adverse events. The higher heart rate and its significant increase at 7‐day ECG could express powerful sympathetic system activation caused by serious infection state or systemic inflammatory response. Furthermore, low QRS voltages may reflect significant lung damage in severe COVID 19 pneumonia and widening of QRS complex during the hospital stay may be an expression of direct heart injury. The increased prevalence of ECG ischemic alterations and elevated baseline cardiac biomarkers (hs‐Tn I) could directly reflect the severe lung parenchymal involvement leading to hypoxemia with a myocardial supply‐demand mismatch or could suggest a COVID‐19‐related myocarditis, as described in some case reports (Doyen et al., 2020; Siripanthong et al., 2020).

Probably, cardiac involvement is the result of different mechanisms, as previously reported for other viruses (Madjid et al., 2019; Sellers et al., 2017). Beyond oxygen supply–demand mismatch, another cause could be the direct protein–receptor interaction. The spike protein of SARS‐CoV‐2 has a strong binding affinity to angiotensin‐converting enzyme 2 (ACE2) receptor, which is also highly expressed in the heart and in the lung parenchyma (Tikellis & Thomas, 2012).

Another putative mechanism is the cytokine storm syndrome with an uncontrolled dysfunctional immune response, leading directly to apoptosis or necrosis of myocardial cells (de Jong et al., 2006; Ruan et al., 2020; Wong et al., 2004). In response to the infection, the acute increase of different circulating pro‐inflammatory cytokines (including IL‐6, IL‐1, TNF‐ α, and interferon) is correlated directly with an unfavorable prognosis in COVID‐19 (Ragab et al., 2020). The cytokine storm can also trigger a prothrombotic and antifibrinolytic imbalance that favors thrombus formation (Libby & Lüscher, 2020). Therefore, antithrombotic therapy with heparin can improve the prognosis of these patients (Paolisso et al., 2020). Finally, inflammatory mediators may also trigger arrhythmias (e.g., atrial fibrillation and/or ventricular arrhythmias), especially in the presence of other precipitating factors (e.g., hypokalemia, hypomagnesaemia, hyperglycemia, or metabolic acidosis) (Kochi et al., 2020; Marfella et al., 2020).

In 7‐day ECG, excessive prolongation of QTc was observed in patients treated with hydroxychloroquine alone or in combination with azithromycin. Regarding the arrhythmic events during the hospitalization, the incidence of new‐onset atrial fibrillation was strictly associated with a higher rate of major adverse events. Only one patient died with a diagnosed third‐degree atrioventricular block. We did not observe any case of sudden cardiac death from proved ventricular arrhythmia. Currently, literature is still lacking about malignant arrhythmias in COVID‐19. In our population, the QTc interval was associated with major adverse events only at ECG at admission and not at 7‐day ECG. Nevertheless, our data demonstrated no association between long QTc interval and ventricular arrhythmias. Our data confirm previous studies in which hydroxychloroquine was not associated with induced ventricular arrhythmias (Gautret et al., 2020; Mercuro et al., 2020; Saleh et al., 2020). In fact, in a prospective study, Chang D. et al found none of 117 COVID‐19 patients treated with hydroxychloroquine had arrhythmias that led to medication discontinuation (Chang et al., 2020).

### 4.2 Study limitations

Our study has some limitations. First, this study was conducted in a single medical center with a relatively small number of patients. Nevertheless, our institution is the regional coordinating center for the COVID‐19 in our region.

Furthermore, in our study, some clinical or laboratory data at baseline and during follow‐up are missing due to practical difficulty in managing clinical reports because of a serious infectious problem. Finally, due to various logistical issues, only 30 echocardiograms were performed, and these data were not analyzed. Indeed, it could be useful to perform an echocardiographic evaluation in each patient with suspected myocardial damage.

## 5 CONCLUSIONS

Our study evaluated the role of serial ECG findings in hospitalized patients with COVID‐19. ECG alterations at admission and even more subsequent ECG findings at 7‐day ECG could help the clinicians stratify the risk of major adverse events in COVID‐19. Serial ECG recordings can track the unfavorable course of patients with COVID‐19. Indeed, ECG alterations were closely linked with the severity of the SARS‐Coronavirus‐2 infection and could express a direct or indirect cardiac involvement related to the physiopathological mechanism of this complex disease.

These findings suggest that in patients with COVID‐19, it is a good practice to collect a basal ECG and repeat the 12 lead ECG evaluation during the hospitalization stay due to the high burden of information related.

## CONFLICT OF INTEREST

None.

## AUTHOR CONTRIBUTIONS

LB, ECD and PP contributed conception and design of the study; MF, FA, FD, ST, IM, LB, AR and CC organized the database; LB and MB performed the statistical analysis; LB wrote the first draft of the manuscript; ECD, PP and ST wrote sections of the manuscript. AR, PV, CP, MB and NG revised the article. All authors contributed to manuscript revision, read and approved the submitted version.

## ETHICAL APPROVAL

Written informed consent was waived by the ethics committee of the designated hospital for patients with emerging infectious diseases.

## Data Availability

The data that support the findings of this study are available from the corresponding author upon reasonable request.
